# Establishment of a Bluetongue Virus Infection Model in Mice that Are Deficient in the Alpha/Beta Interferon Receptor

**DOI:** 10.1371/journal.pone.0005171

**Published:** 2009-04-09

**Authors:** Eva Calvo-Pinilla, Teresa Rodríguez-Calvo, Juan Anguita, Noemí Sevilla, Javier Ortego

**Affiliations:** 1 Centro de Investigación en Sanidad Animal, CISA-INIA, Madrid, Spain; 2 Department of Veterinary and Animal Sciences, University of Massachusetts Amherst, Amherst, Massachusetts, United States of America; University of California, San Francisco, United States of America

## Abstract

Bluetongue (BT) is a noncontagious, insect-transmitted disease of ruminants caused by the bluetongue virus (BTV). A laboratory animal model would greatly facilitate the studies of pathogenesis, immune response and vaccination against BTV. Herein, we show that adult mice deficient in type I IFN receptor (IFNAR^(−/−)^) are highly susceptible to BTV-4 and BTV-8 infection when the virus is administered intravenously. Disease was characterized by ocular discharges and apathy, starting at 48 hours post-infection and quickly leading to animal death within 60 hours of inoculation. Infectious virus was recovered from the spleen, lung, thymus, and lymph nodes indicating a systemic infection. In addition, a lymphoid depletion in spleen, and severe pneumonia were observed in the infected mice. Furthermore, IFNAR^(−/−)^ adult mice immunized with a BTV-4 inactivated vaccine showed the induction of neutralizing antibodies against BTV-4 and complete protection against challenge with a lethal dose of this virus. The data indicate that this mouse model may facilitate the study of BTV pathogenesis, and the development of new effective vaccines for BTV.

## Introduction

Bluetongue (BT) is an infectious noncontagious viral disease of ruminants caused by the bluetongue virus (BTV). The virus, consisting of 24 different serotypes, is transmitted to its vertebrate host by a few species of biting midges of the *Culicoides* genus (*Diptera*: *Ceratopogonidae*) [1]. BT is a reportable disease of considerable socioeconomic concern and of major importance in the international trade of animals and animal products. From 1998 through 2005, at least 6 BTV strains belonging to 5 serotypes (BTV-1, BTV-2, BTV-4, BTV-9, and BTV-16) were continously present in the Mediterranean Basin [2], [3], [4]. Since August 2006, BTV-8 has caused severe epizootic outbreakes in northern Europe [5]. The emergence of BT in parts of Europe never before affected was attributed mainly to climate change and linked to the Northern expansion of the major Old World vector *Culicoides imicola*. Additionally, there is also evidence for the involvement of other novel indigenous European vector species of *Culicoides* (*C. obsoletus* and *C. pulicaris* ) [6].

BTV has a genome composed of ten linear segments of double-stranded RNA (dsRNA) and is classified as the type species of the genus *Orbivirus* within the family *Reoviridae* [7]. The BTV genome encodes 7 structural (VP1 through VP7) and 4 nonstructural proteins (NS1 through NS3/NS3A). The outer capsid is composed of two major structural proteins, VP2 and VP5 (segments 2 and 6, respectively), involved in cell attachment and virus entry. VP2 is known to contain the major neutralization determinant of BTV while VP5 influences virus neutralization through its conformational interaction with VP2 [8]. The outer capsid covers the inner capsid that is composed of two major structural proteins VP3 and VP7 (encoded by segments 3 and 7, respectively) and three distinct minor proteins (VP1, VP4, and VP6 corresponding to segments 1, 4, and 9 respectively) [9], in addition to the viral genome. Four other nonstructural proteins, produced during the viral cycle (NS1, NS2, and NS3/NS3A) (segments 6, 8, and 10 respectively), are more conserved among serotypes [10].

Studies involving the natural hosts of BTV are limited by the complexity of the system, the scarce knowledge of their immune system, and the need to have an animal facility with biosafety level 3. To circumvent some of these problems, an adequate system would be the use of adult mice because of the knowledge of its genetics and its manageability. BTV infects newborn mice [11], [12], [13], but an adult mouse model will be necessary to allow studies of acquired immune responses and vaccination against BTV.

Bluetongue virus is a potent interferon alpha (IFN-α) inducer [14], [15], [16]. In addition, a temporal relationship between viremia and IFN-α activity has been observed in sheep infected with BTV, where IFN peak concentrations induced approximately a 90% decrease in virus titer [17]. IFN-α plays an essential role in the antiviral innate immune response. Virus-derived dsRNAs are detected by Toll-like receptors on type I IFN-producing cells (mostly notably on plasmacytoid dendritic cells). Secreted IFNs bind to the type I IFN receptor (IFNAR) on the surface of neighbouring cells, activating the Janus kinase (Jak)/signal transducer and activator of transcription (Stat) signalling pathway. This in turn induces the transcriptional activation of target genes, the products of which render the cells resistant to virus replication through various mechanisms, including degradation of viral mRNAs, inhibition of viral translation, and inhibition of cell growth [18], [19], [20], [21]. Blocking IFN-α/β activity in mice leads to a dramatically increased sensitivity to many viruses. Genetically targeted (knockout) mice lacking the β subunit of the IFN-α/β receptor (IFNAR^(−/−)^ mice) are unable to establish an antiviral state and, as a consequence, are highly susceptible to many viral infections, despite the presence of an otherwise intact immune system [20], [22]. Recently, Ida-Hosonuma et al [23] found that the deletion of the IFNAR gene in the IFNAR^(−/−)^ mice resulted in the disruption of IFN- α/β-induced signaling, which is an important determinant of the tissue tropism and pathogenicity of poliovirus. Similarly, it has been reported that IFN- α/β plays an important role in the pathogenicity and tissue tropism of some viruses. The lack of an IFN system allows the virus to replicate more efficiently and IFNAR^(−/−)^ mice have been used as a laboratory animal model to study the immune response and pathogenicity of coronaviruses, vaccinia virus Ankara strain (MVA), measles virus, Rift valley fever virus and West Nile virus [24], [25], [26], [27]. All these data, and the presence of an otherwise intact immune system in these mice [20], [22] suggest that IFNAR^(−/−)^ mice could be a good animal model to study BTV infections and to evaluate vaccine strategies against this virus.

Here, we report the establishment of a new laboratory animal model suitable for the evaluation of vaccination strategies against BTV. Adult IFNAR^(−/−)^ mice support the *in vivo* growth of BTV-4 after intravenous inoculation. BTV-4 replicated in spleen, lung, thymus, and lymph nodes (popliteal, inguinal, mediastinal, and mesenteric) of IFNAR^(−/−)^ mice reproducing the tropism observed during calf and sheep infections. In addition, IFNAR^(−/−)^ mice vaccinated with a BTV-4 inactivated vaccine show complete protection against a lethal dose of BTV-4

## Results

### BTV-4 causes a lethal infection in adult IFNAR^(−/−)^ mice

**Figure 1:**
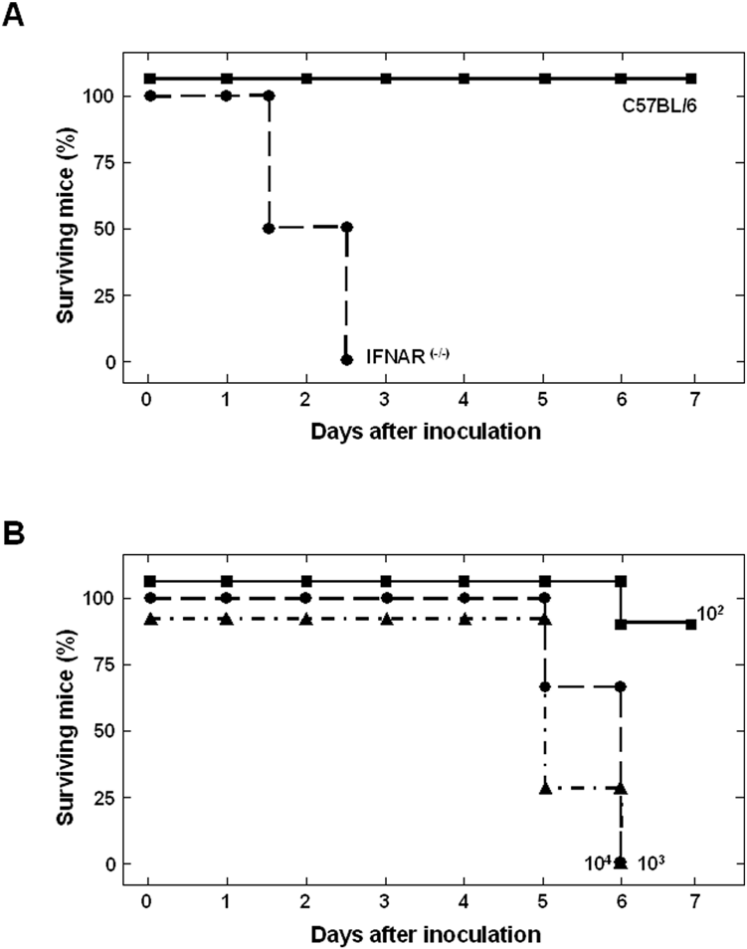
Susceptibility of adult mice to BTV-4 infection. (A) C57BL/6 and IFNAR^(−/−)^ mice (8 weeks old, 6 mice per group) were intravenously inoculated with 10^6^ PFUs of BTV-4. The mice were observed every 12 h for the first 3 days and every 24 h for 7 days. (B) Survival rates of IFNAR^(−/−)^ mice after inoculation with BTV-4. Mice (8 weeks old, 6 mice per group) were intravenously inoculated with 10-fold dilutions of BTV-4. The number of PFUs inoculated is indicated on each survival group. The mice were observed every 24 h for 7 days.

In order to develop an adult murine model for BTV infection in which mice showed disease symptoms, we tested the susceptibility of C57BL/6 and IFNAR^(−/−)^ mice to BTV infection. Since blood is the natural route of BTV infection in ruminants, adult C57BL/6 and IFNAR^(−/−)^ mice (males, 8 weeks old) were infected intravenously (i.v.) with 10^6^ PFUs of BTV-4. Under these conditions, C57BL/6 mice did not show any disease symptom or death following viral infection. By contrast, IFNAR^(−/−)^ mice were susceptible to BTV-4 infection (Fig. 1A), showing disease symptoms characterized by ocular discharges and apathy starting at 48 h.p.i. Disease progression led to animal death within 60 h.p.i. The LD_50_ value was obtained by i.v. inoculation with 10-fold dilutions of BTV-4, resulting in a LD_50_ value of 10^2.6^ PFU (Fig. 1B). At low infectious doses (10^2^ PFU or less) mice survived up to 21 days, at which point the experiment was terminated (data not shown).

**Figure 2:**
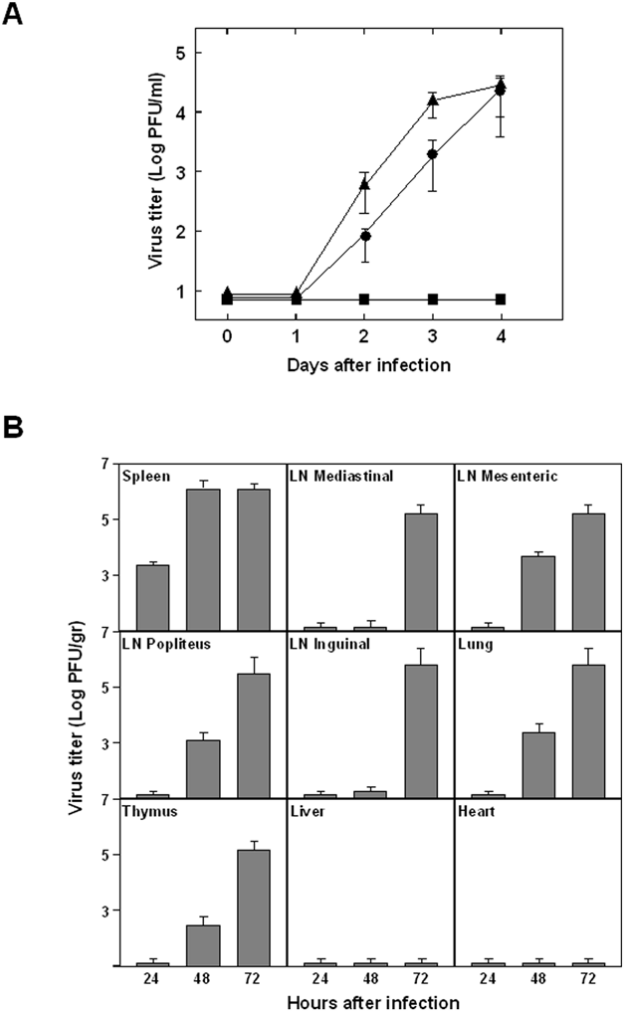
BTV-4 titers in blood and several organs of infected IFNAR^(−/−)^ mice. (A) Titers of BTV-4 recovered in blood after intravenous infection with 10^2^ (▪), 10^3^(•), or 10^4^ (▴) PFUs of BTV-4. Virus was extracted from blood and determined as described in Materials and Methods. Each point represents the mean values of the viral titer of six animals, and standard deviations are shown as error bars. (B) Mice (8 weeks old, 6 mice per group) were inoculated intravenously with 10^4^ PFUs of BTV-4. Virus was extracted from the indicated tissues at 24, 48 and 72 hours after infection for virus titration. Standard deviations are given. Procedures are detailed in Materials and methods.

To determine virus dissemination, viral titers in blood samples after i.v. inoculation with 10^4^ PFUs of BTV-4 were analyzed. According to the previous data, no viremia was detected in C57BL/6 mice (data not shown). In contrast, viremia was observed in IFNAR^(−/−)^ mice at day 2 post-infection (Fig. 2A), with peak titers of 5×10^4^ PFU/ml at day 4 post-infection (p.i.), before animal death. IFNAR^(−/−)^ mice inoculated with 10^2^ PFU did not show any viremia but titers up to 3×10^4^ PFU/ml were observed at 3 and 4 days p.i. in mice inoculated with 10^3^ PFUs. Viral spread was determined in tissue samples. The first tissue to be reached by the virus was the spleen (Fig. 2B) where infectious virus was detected as early as 24 h.p.i. (5×10^3^ PFU/gr), with viral titers increasing thereafter until death (reaching titers of 2×10^6^ PFU/gr). By 48 h.p.i. significant titers of BTV-4 were detected in spleen, lung, thymus, and popliteal and mesenteric lymph nodes. At 72 h.p.i., titers up to 10^6^ PFU/gr of BTV-4 were recovered from the spleen, lung, thymus, and lymph nodes (popliteal, inguinal, mediastinal and mesenteric). No infectious virus was detected in liver, brain, heart, tongue, skin, and testicles at any time points examined, even when the tissues were analyzed by RT-PCR (data not shown). Interestingly, the virus was not detected in the blood until 48 h.p.i. These results suggest that the virus leaks into the blood stream after replicating in the spleen.

### Susceptibility of IFNAR^(−/−)^ mice to other BTV serotypes

**Figure 3:**
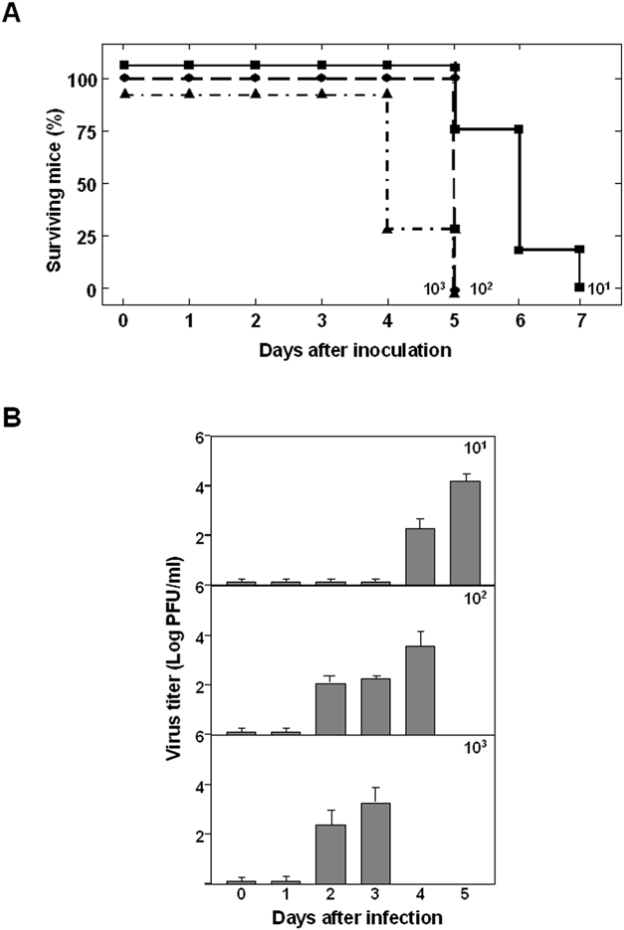
Survival rates of IFNAR^(−/−)^ mice and virus titers in blood after inoculation with BTV-8. (A) Mice (8 weeks old, 6 mice per group) were intravenously inoculated with 10-fold dilutions of BTV-8. The number of PFUs inoculated is indicated on each survival group. The mice were observed every 24 h for 7 days. (B) Titers of BTV-8 recovered in blood after intravenously infection with 10-fold dilutions of BTV-4. The number of PFUs inoculated is indicated on each survival group. Virus was extracted from blood and determined as described in Materials and Methods. Each point represents the mean values of the viral titer of six animals, and standard deviations are shown as error bars.

To determine whether IFNAR^(−/−)^ mice were susceptible to BTV of different serotypes, IFNAR^(−/−)^ mice were inoculated with ten-fold serial dilutions of BTV-8. Mice infected with serotype 8 showed similar symptoms to those shown after infection with serotype 4. However, BTV-8 showed higher virulence than BTV-4 with only 10 PFUs of BTV-8 being enough to kill 100% of the mice at day 7 p.i. (Fig. 3A), while BTV-4 was not lethal at the same infective dose (Fig. 1B). The viremia was determined after i.v. inoculation with 10-fold dilutions of BTV-8 (Fig. 3B). BTV-8 was first detected in blood of mice inoculated with 10 PFUs at day 4 post-infection, and the titer increased up to 2×10^4^ PFU/ml at day 5 p.i. Titers up to 8×10^3^ PFU/ml were observed at 3 and 4 days p.i. in mice infected with 10^3^ and 10^2^ PFUs, respectively. In the three dilutions of virus analyzed, viral titers increased thereafter until animal death. Although BTV-8 showed higher virulence than BTV-4 in IFNAR^(−/−)^ mice, BTV-8 and BTV-4 titers in blood were equivalent, indicating that the higher virulence of serotype 8 is not due to a higher level of viral replication. These data suggest that IFNAR^(−/−)^ mice could be an adequate small animal model to study differences in virulence among BTV serotypes.

### BTV-4 infection of IFNAR^(−/−)^ adult mice causes microscopic lesions in several tissues

**Figure 4:**
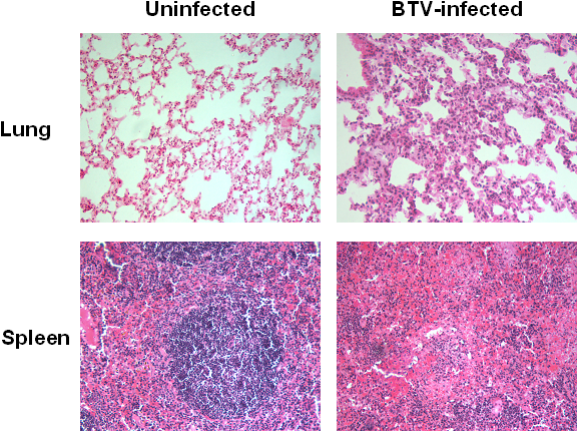
Tissue sections of lung and spleen from BTV-4 infected adult IFNAR^(−/−)^ mice. Mice were infected with 10^3^ PFUs of BTV-4 intravenously. Tissues were harvested at 48 h.p.i.. Hematoxylin and eosin staining are shown. Lungs of BTV-infected mice show hyperemia and increased size of the interalveolar septa (100×). The spleen sections of BTV-infected mice show loss of architectural structure and marked lymphoid depletion (100×).

In order to study the pathological effects of the infection in the organs where BTV replicates, histological analysis were performed on material of several organs extracted from BTV infected and uninfected IFNAR^(−/−)^ mice at 48 h.p.i. Gross pathological alterations were characterized by widespread oedema, haemorrhages especially in spleen and lungs, and enlarged spleen and lymph nodes. Histological examination of lungs of BTV infected mice showed hyperemia and increased septum size with infiltration of lymphocytes, inactivated macrophages and a few neutrophils (Fig. 4). The peribronchial and perivascular connective tissue contained a few infiltrating round cells (Fig. 4). These histopathological findings are consistent with bronchointerstitial pneumonia. There was also a moderate oedema in the alveolar cavity with presence of abundant alveolar macrophages and a few detached epithelial cells (descamative alveolitis). The infected spleen showed a marked lymphoid depletion with infiltration of neutrophils in the white pulp (Fig. 4). This lymphoid depletion was also observed in the thymus as well as the loss of thymic architecture with the medulla and the cortex becoming not distinguishable (data not shown). The results suggest that BTV-4 produces similar tissue lesions in IFNAR^(−/−)^ mice than in the natural host.

### Immunized IFNAR^(−/−)^ mice are completely protected against a lethal BTV-4 challenge

**Figure 5:**
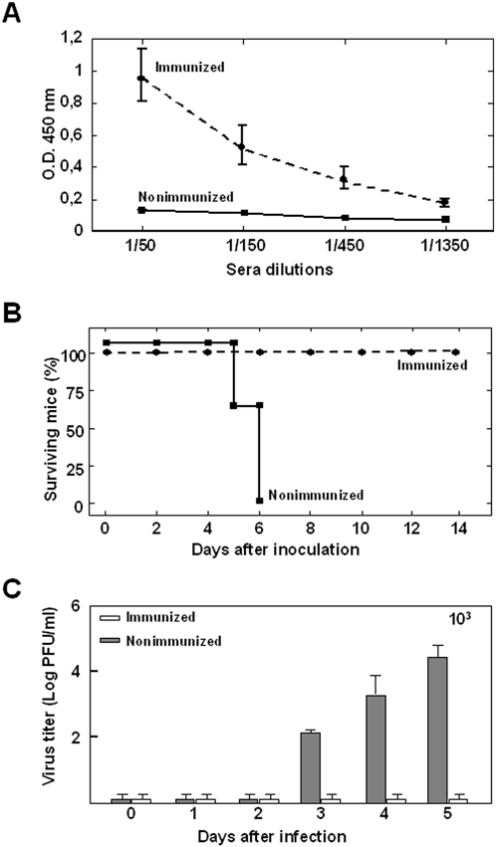
Protection of vaccinated IFNAR^(−/−)^ mice against a lethal BTV-4 challenge. Mice (8 weeks old, 8 per group) were immunized twice by subcutaneous administration of an inactivated BTV-4 vaccine and inoculated with 10^3^ PFUs of BTV-4,. (A) ELISA detection of antibodies to BTV-4 VP2 in serum of immunized and nonimmunized IFNAR^(−/−)^ mice. Serum was collected two days before the challenge with BTV-4, and dilutions (1∶50 to 1∶1350) were analyzed by ELISA as described in Materials and Methods. (B) Survival rates of immunized and nonimmunized IFNAR^(−/−)^ mice after inoculation with BTV-4. The mice were observed every 24 h for 14 days. (C) Titers of BTV-4 recovered in blood of immunized and nonimmunized IFNAR^(−/−)^ mice after challenge with BTV-4. Virus was extracted from blood and determined as described in Materials and Methods. Each point represents the mean values of the viral titer of eight animals, and standard deviations are shown as error bars.

All the previous data indicated that BTV-4 caused a lethal infection in adult IFNAR^(−/−)^ mice. To provide further proof that BTV-4 was the causative agent of the disease and death in these animals and that IFNAR^(−/−)^ mice are a good animal model for BTV vaccination studies, vaccination protection experiments were performed. Adult IFNAR^(−/−)^ mice were immunized with a ZULVAC-BTV-4 inactivated BTV-4 preparation. The vaccine was administered by two consecutive subcutaneous injections of the equivalent to 3×10^5^ TCID_50_ of BTV-4 at 3 weeks intervals. After the second immunization, VP2 antibody titers were determined by ELISA. All immunized animals, but not the control animals, developed an antibody response (Fig. 5A) indicating a successful immunization. In addition, the immunization induced neutralizing antibodies against BTV-4 in IFNAR^(−/−)^ mice (VNT 1.53±0.32) as detected by virus neutralization tests. Three weeks after the second immunization, immunized and control IFNAR^(−/−)^ mice were challenged intravenously with 10^3^ PFUs of BTV-4. While all nonimmunized animals died, 100% of the immunized animals were protected against a lethal challenge (Fig. 5B). Infectious viral titers were analyzed in the blood of immunized and nonimmunized IFNAR^(−/−)^ mice by plaque assay after intravenous infection with BTV-4 (Fig. 5C). No Infectious viruses were detected in immunized mice; however, we observed titers up to 3×10^4^ PFU/ml in nonimmunized animals at day 5 post-challenge. In addition, we analyzed by RT-qPCR the presence of BTV genomes in the blood of inmmunized and nonimmunized IFNAR^(−/−)^ mice challenged with BTV-4. The results are summarized in Table 1. BTV genomes were readily detected in nonimmunized mice at days three and four after BTV infection (C*t*: 27–29) and increased (C*t*: 23–26) thereafter until the death of the animal. In contrast, the RT-qPCR reaction yield no positive results for the majority of the immunized mice (n = 5) at all days post-challenge analyzed (*C*t≥38). Viral genomes were detected in one of the vaccinated mice (C*t*: 30) at days four and five post-challenge and in two of them at day 5 (C*t*: 32). However, in these three immunized mice the C*t* was higher than in the nonimmunized mice and the presence of BTV-genomes reverted to negative at day 7 post-challenge. Overall, these data indicate that protective immunity was achieved after vaccination. These results confirm that BTV-4 was the causative agent of disease and death of adult IFNAR^(−/−)^ mice.

**Table 1:** Detection of BTV-4 in blood of immunized and nonimmunized IFNAR^(−/−)^ mice after challenge with BTV-4 by RT-qPCR_S5.

Animals	Days post-challenge				
	3	4	5	7	10
**C-1**	29.07	25.05	†		
**C-2**	neg.	27.38	23.78	†	
**C-3**	29.75	26.80	†		
**C-4**	neg.	27.57	23.45	†	
**I-1**	neg.	neg.	neg.	neg.	neg.
**I-2**	neg.	30.57	30.74	neg.	neg.
**I-3**	neg.	neg.	neg.	neg.	neg.
**I-4**	neg.	neg.	neg.	neg.	neg.
**I-5**	neg.	neg.	neg.	neg.	neg.
**I-6**	neg.	neg.	neg.	neg.	neg.
**I-7**	neg.	neg.	31.94	neg.	neg.
**I-8**	neg.	neg.	32.06	neg.	neg.

Results expressed as *C*t and transferred to negative (neg.) according to the cut-off *C*t≥38 described by Toussaint et al. (2007).

I, immunized mice. C, nonimmunized mice.

† , death mice.

## Discussion

For years, different groups have tried to establish a laboratory animal model to facilitate the studies of pathogenesis, immune response and vaccination against BTV. Natural hosts, although excellent tools for studies of pathogenesis and vaccination, are expensive and required specialized laboratories. Here, we characterize a new animal model based on adult IFNAR^(−/−)^ mice that support the *in vivo* growth of BTV-4 and BTV-8 after intravenous inoculation. A tremendous advantage of this mouse model is the availability of a wide variety of reagents that can be used to study many aspects of the immune response to the virus. In addition, we propose this animal model as an adequate system for testing BTV vaccines, an important issue because the cost of testing new vaccines in target species is a major obstacle for laboratories and industries. The new mouse model for the study of BTV infection has unique features that open the possibility to study in the same host-virus system susceptibility, virulence, immunobiology of infection, and vaccine efficacy, in ways that are not approachable with the natural host.

BTV infects newborn mice [11], [12], [13], [28], but an adult animal model will be necessary to allow studies of acquired immune responses and vaccination against BTV. Different approaches have been followed to avoid this problem. The protective effects of baculovirus expressed BTV-10 minor and non-structural proteins were assessed by measuring virus titers in the ovaries of mice challenged with homologous recombinant vaccinia virus (rVV). Protection mediated by CTLs specific for the BTV minor or nonstructural proteins could not be evaluated by challenging mice with BTV-10, as the virus does not cause disease in adult mice [29]. The infection of fetal mice with BTV leads to severe cerebral malformation. Infection of 2-week-old mice resulted in very limited multiplication without sequelae, and infection of 4-week-old mice did not show clinical disease and no viral multiplication was detected [30]. Thus, the use of highly immature mice results in pathologies that do not resemble those found in the natural host. Another aspect to consider is the route of inoculation that may determine the outcome of infection. In this work, we have used the intravenous inoculation to resemble the natural infection route by mosquito biting. Thus, mice infected with BTV i.v. develop clinical symptoms, as it happens in natural hosts. This makes the mouse model even more atractive to be used as a tool for studying immune response to BTV and vaccination strategies.

Up-regulation of type I IFN is one of the earliest cellular responses upon contact with infectious agents. The rapid induction of type I IFN reflects the crucial role that this cytokine plays in the inhibition of viral spread before the generation of a specific immune response. Given that viruses must at least partially circumvent the IFN response, it is not surprising that an inability to do this can restrict host range. There are many examples where this is known. For example, neither measles virus nor polio virus will replicate efficiently in mouse models unless the IFN system has been compromised [24], [31]. BTV is a potent type I interferon inducer in mice and cattle [14], [16], [32]. Our results show that BTV is pathogenic for IFNAR^(−/−)^ mice mainly due to the lack of the IFN-I receptor, allowing the virus to circumvent the IFN-I response in the host. In addition, IFNAR^(−/−)^ mice serve as a good animal model for BTV, reproducing many aspects of its natural host infection. First, IFNAR^(−/−)^ mice infected with BTV showed viremia and disease symptoms, in contrast with C57BL/6 mice, which did not show them, even when these animals were infected at the highest viral dose tested (10^6^ PFUs/mouse). The appearance of viremia after inoculation in infected IFNAR^(−/−)^ mice was dependent on the viral dose, althought the highest titers observed in blood were not dose-dependent. Second, the differential virulence of serotypes 4 and 8 were maintained in this animal model. Some BTV serotypes such as serotype 8, which recently caused infection in northern Europe, exhibit enhanced virulence in cattle [5], in contrast to BTV-4 that usually exhibits subclinical infections in this species. In infected IFNAR^(−/−)^ mice, BTV-8 killed 100% of the animals with a dose as low as 10 PFUs per mouse. In contrast 10^3^ PFUs of BTV-4 were needed to kill 100% of the mice. In addition, the virus titers found in the blood of infected mice were similar at the same virus doses for the animals infected with BTV-8 than with BTV-4, indicating that BTV-8 exhibited a higher virulence than BTV-4 in the IFNAR^(−/−)^ model, as is observed in cattle, one of the BTV natural hosts. Third, BTV dissemination in IFNAR^(−/−)^ mice reproduced BTV infection in cattle and sheep. High titers of BTV are present in the lungs, precapsular and mesenteric lymph nodes, thymus, and spleen of infected calves [33], [34]. In IFNAR^(−/−)^ mice, the virus was detected in spleen before was isolated from other organs, including blood. Release of BTV from spleen was followed by an increased in viremia and dissemination to other organs (lymph nodes, lung and thymus), followed by its dissemination to lymph nodes, lung, and thymus after intravenous infection. This has been suggested also for calves [34] indicating a similar BTV spreading pattern in IFNAR^(−/−)^ mice. Histological analyses of BTV infected mice at 48 h.p.i. showed many similarities with lessions described in natural hosts. The permeability disorders of the vascular system described for ruminants [35] is reflected in the petecheias observed in spleen in BTV infected mice. The enlargement of lymph nodes reflects an ongoing immune response. However, the observed lymphoid depletion suggests that other cells are migrating to these organs or that a general aedema results in organ enlargement. The lungs are especially susceptible to permeability disorders of the vasculature induced by BTV, and this is consistent in BTV infected mice as well as in ruminants. In general, pathology in natural hosts has not been profoundly studied. The described pathology similarities between IFNAR^(−/−)^ mice and ruminants after infection with BTV indicate that our mouse model may be a good tool to study new findings in BTV pathology.

The cost of testing new vaccines in target species is a major obstacle for laboratories and industries. For this reason, the intracerebral inoculation of newborn mice with BTV vaccines has been used as an animal model to evaluate the level of attenuation of live attenuated BTV vaccines [28]. Our results show that adult IFNAR^(−/−)^ mice serve as a good animal model to test BTV vaccines. Even though the lack of type I interferon signals may have an effect in the development of acquired immune responses, our results and previous studies [20], [22], [24], [25], [26], [27] show that the IFNAR^(−/−)^ infection model is useful for the definition of effective vaccine candidates against BTV. Indeed, in our study all immunized animals developed an antibody response specific of BTV-4 with the production of neutralizing antibodies against the same serotype, indicating a successful immunization. The protection mediated by inactivated whole BTV vaccine in IFNAR^(−/−)^ mice infected with a lethal dose of BTV-4 was complete, and 100% of the animals did not show any symptoms associated with infection or died. Viremia was not detected in the blood of immunized animals after challenge with BTV-4 when analyzed by plaque assay. The presence of BTV genomes in the blood of immunized mice after BTV-4 challenge analyzed by RT-qPCR, a more sensible method than the plaque assay, showed the presence of viral genomes in the blood of some of the immunized mice but in all the cases, the *C*ts were higher than in the nonimmunized mice and those that were RT-qPCR positive reverted to negative at day seven post-challenge. Similar RTqPCR results have been observed in cattle and sheep vaccinated with ZULVAC-BTV-4 inactivated BTV-4 preparation [36] These results strongly support the involvement of BTV in the pathological processes described in this work and, moreover, suggest that the mouse model is adequate to evaluate vaccine candidates. Future work will determine whether the ability to protect mice with other vaccine formulations mimics the capacity to protect the natural host.

In summary, we have characterized a novel small animal model for BTV infection based on IFNAR^(−/−)^ adult mice that reproduces many aspects of its natural host infection. This animal model may facilitate the understanding of the mechanisms of BTV pathogenicity in its natural host and a faster advance in the development of new BTV vaccines.

## Materials and Methods

### Virus and cells

Baby hamster kidney cells (BHK-21), and Vero cells were grown in Dulbecco's modified Eagle's medium (DMEM) supplemented with 2mM glutamine and 10% fetal calf serum (FCS). Standard virus titrations were performed in Vero cells. BTV serotypes 4 (Spain/01) and 8 (Belgium/06), originally isolated from an infected sheep in Spain in 2001 and a calf in Belgium in 2006, respectively, were used in the experiments. Virus stocks were generated by infection of confluent BHK-21 cells using a multiplicity of infection (MOI) of 1. At 48 h.p.i. or when total cytopathic efect (CPE) was visible, the cells and supernatants were harvested and centrifuged. The virus was released from the cells by three freeze and thaw cycles.

### Mice

C57BL/6 mice were purchased from Harlan Interfauna Ibérica S.L. IFN α/βR^o/o^ IFNAR^(−/−)^ mice [20], on a C57BL/6 genetic background, were generously provided by Professor R. Zinkernagel (Institute of Experimental Medicine, Zurich). All mice used were matched for sex and age (males 8 weeks). Mice were maintained under pathogen-free conditions and allowed to acclimatize to the biosafety level 3 (BSL3) animal facility at the Centro de Investigación en Sanidad Animal, INIA, Madrid, for 1 week before use in our experiments. All experiments with live animals were performed under the guidelines of the European Community (86/609) and were approved by the site ethical review committee.

### Animal inoculation and processing of samples

Mice were infected intravenously with different doses of virus. Mice were examined for clinical symptoms daily. LD50 values were calculated by the method of Reed and Muench (1938) [37], after inoculation of mice with 10-fold serial dilutions of virus (from 10^6^ to 10^1^ PFU/mouse). Whole blood was collected in EDTA from all animals at regular intervals after inoculation. At varying times post-infection, several mice were sacrificed by perfusion with phosphate-buffered saline (PBS), and several organs (spleen, lung, thymus, liver, brain, heart, tongue, skin, and testicles), and lymph nodes (popliteal, inguinal, mediastinal, and mesenteric) were harvested. Tissues were homogenized in PBS using a Tissue Lyser homogenizer (Quiagen). The viruses were released from whole blood and homogenized tissues by three freeze/thaw cycles. The amount of infectious virus was measured by plaque assay on Vero cells.

### Murine immunizations

Groups of 6 IFNAR^(−/−)^ mice were immunized by two consecutive subcutaneous injections of either ZULVAC-BTV-4 (1.5×10^6^ TCID_50_BTV-4) inactivated BTV-4 preparation (Fort Dodge Veterinaria, S.A.) or phosphate-buffered saline (PBS) (controls), administered 3 weeks apart. Mice were intravenously inoculated with 10^3^ PFUs of BTV-4 (lethal dose) 3 weeks after the last immunization. Mice were bled before each immunization and virus challenge. Sera were tested for BTV-4 neutralizing antibodies by Virus Neutralization Test (VNT).

### Histopathology

Samples from different tissues and organs were taken and fixed in 10% buffered formalin (pH 7.2) for histopathological studies. After fixation, samples were dehydrated through a graded series of alcohol to xylol and embedded in paraffin wax. Sections of 4-im-thick were cut and stained with hematoxylin and eosin (H & E) for histopathological analyses.

### BTV-VP2 antibody detection by indirect ELISA

MaxiSorp plates (Nunc, USA) were coated with VP2 baculovirus expressed protein (164 ng per well) and incubated overnight at 4°C. Plates were saturated with blocking buffer (PBS-0.05% Tween 20 and 5% skimmed milk). The animal sera, diluted in blocking buffer were added and incubated for 1 hour at 37°C. After three washes in PBS-0.05% Tween 20, plates were incubated for 1 hour at 37°C with an anti-mouse-HRP secondary antibody (Biorad, USA) at a 1/2,000 dilution in blocking buffer. Finally, after three washes in PBS-0.05% Tween 20, the reaction was developed with 50 µl of substrate solution 3,3′, 5,5′–tetramethylbencidine liquidsupersensitive (TMB) (Sigma) and stopped by adding 50 µl of 3N H_2_SO_4_. Results were expressed as optical densities (ODs) measured at 450 nm.

### BTV-4 neutralizing antibody detection in immunized mice by virus neutralizing test (VNT)

The VNT was used to determine neutralizing antibody titers against BTV-4. For plaque reduction assays, 2 fold dilutions of sera were mixed with 100 PFU of BTV-4, incubated for 1 hour at 37°C and then plated onto monolayers of Vero cells. After 1 hour, agar overlays were added and the plates were incubated for 5 days. The titer was determined as the highest dilution that reduced the number of plaques by 50%.

### RT-qPCR specific for BTV segment 5

Whole blood was collected in EDTA from all animals at regular intervals after inoculation and BTV challenge. Total RNA was extracted from blood with TRI Reagent Solution (Ambion), according to the method recommended by the manufacturer. The real-time RT-qPCR specific for BTV segment 5 was performed as described by Toussaint et al. (2007) [38].
